# mRNA Extraction from Gill Tissue for RNA-sequencing

**DOI:** 10.21769/BioProtoc.3539

**Published:** 2020-03-05

**Authors:** Jukka-Pekka Verta, Felicity Jones

**Affiliations:** 1 Organismal and Evolutionary Biology Research Programme, University of Helsinki, Helsinki, Finland; 2 Friedrich Miescher Laboratory of the Max Planck Society, Tuebingen, Germany

**Keywords:** Transcriptome sequencing, Gene regulation, Gene expression evolution, Direct mRNA extraction, Gills

## Abstract

Adaptation is thought to proceed in part through spatial and temporal changes in gene expression. Fish species such as the threespine stickleback are powerful vertebrate models to study the genetic architecture of adaptive changes in gene expression since divergent adaptation to different environments is common, they are abundant and easy to study in the wild and lab, and have well-established genetic and genomic resources. Fish gills, due to their respiratory and osmoregulatory roles, show many physiological adaptations to local water chemistry, including differences in gene expression. However, obtaining high-quality RNA using popular column-based extraction methods can be challenging from small tissue samples high in cartilage and bone such as fish gills. Here, we describe a bead-based mRNA extraction and transcriptome RNA-seq protocol that does not use purification columns. The protocol can be readily scaled according to sample size for the purposes of diverse gene expression experiments using animal or plant tissue.

## Background

Transcriptome sequencing (RNA-seq) is used to quantify the expression levels of genes, to identify differences in gene expression levels between groups of samples and to infer gene co-expression. In evolutionary genetics research, RNA-seq can be used as an approach to study the molecular basis of adaptive divergence (*e.g.*, Rougeux *et al.*, 2019; Verta and Jones, 2019), identify candidate genes underlying adaptive phenotypes (*e.g.*, Ferreira *et al.*, 2017), and infer function for unknown genes (*e.g.*, Rawat *et al.*, 2015) among other applications. A key strength of RNA sequencing is that it can be readily applied to almost any species or tissue, and transcriptome sequencing has accelerated evolutionary research by opening non-model species for genomic studies. Obtaining high-quality mRNA is the most important prerequisite for a successful RNA-seq study (Gallego Romero *et al.*, 2014). This can be challenging because RNA is inherently more unstable (and fragile) than more commonly studied DNA. Standard RNA extraction methods typically yield total RNA, comprising mRNA destined to be translated into proteins, as well as non-translated RNA such as ribosomal and regulatory RNAs. Typically, only 1-4% of total RNA is useful for mRNA sequencing and so mRNA yield from small amounts of tissue can be low. In addition, heterogenous tissue samples such as fish gills that consists of both soft epithelia, cartilage and bone, as well as DNA-rich tissue samples, can perform sub-optimally in column-based RNA extraction methods because of physical clogging of column pores and/or DNA precipitation. In such cases direct extraction of mRNA from tissue lysates using oligo-dT coated magnetic beads offer a straightforward and scalable solution for extraction.

 Here, we describe such bead-based protocol for rapid (~1-2 h) mRNA extraction from fish gills and RNA-sequencing. The oligo-dT based technique relies on A-T hybridization of mRNA poly-A tails with T-oligonucleotide fragments covalently attached to magnetic beads, allowing for full-length mRNA purification from crude cell lysates. This protocol has been adapted from the original protocol for oligo-dT Dynabeads from Invitrogen and was briefly described in Verta and Jones (2019). Slightly different versions of this protocol were previously used to study gene expression in small plant tissues that comprise of ~1,000-2,000 cells (Verta *et al.*, 2016; Ojeda *et al.*, 2019). Briefly, tissue samples are homogenized and lysed, after which mRNA is captured from tissue lysate using magnetic oligo-dT beads. The bead-mRNA complex is washed and subsequently eluted with water. The protocol yields high-quality mRNA that can be used for applications such as RNA-seq and qPCR. We further provide an example of the steps involving RNA quality-control and quantification, RNA-seq library preparation and data analysis. For general guidelines in RNA-seq study design and best-practices the reader is referred to Conesa *et al.* (2016).

## Materials and Reagents

1. 1.5 ml RNase-free Safe-Lock Eppendorf tubes
2. 21 G needles
3. Micro-Tube polypropylene pestles for 1.5 ml Eppendorf tubes (Bel-Art, catalog number: BAF199230001)
4. Qubit Assay tubes (Thermo Fisher, catalog number: Q32856)
5. RNase-free PCR strips and caps
6. Fresh tissue or liquid nitrogen snap-frozen tissue
7. Tricaine methanesulfonate (MS-222, Sigma-Aldrich, catalog number: E10521)
8. Sodium bicarbonate NaHCO_3_ (Sigma-Aldrich, catalog number: S5761)
9. Dynabeads mRNA direct purification kit (Thermo Fisher Invitrogen, catalog number: 61011)
10. Turbo DNase-free kit (Thermo Fisher Invitrogen, catalog number: AM1907)
11. Bioanalyzer RNA 6000 Nano kit (Agilent, catalog number: 5067-11511)
12. Bioanalyzer High Sensitivity DNA kit (Agilent, catalog number: 5067-4626)
13. Qubit RNA BR reagents (Thermo Fisher, catalog number: Q10211)
14. PrimeScript RT Master Mix (Takara, catalog number: RR036A)
15. Sybr Select Master Mix for CFX (Thermo Fisher Applied Biosciences, catalog number: 4472942)
16. TruSeq stranded mRNA kit (Illumina, catalog number: 20020594)
17. SuperScript II reverse transcriptase (Thermo Fisher, catalog number: 18064014)

## Equipment

1. Scissors (or a blade) and forceps
2. DynaMag2 magnet (Thermo Fisher Invitrogen, catalog number: 12321D)
3. PCR cycler (*e.g.*, Bio-Rad C1000 Touch, catalog number: 1851196)
4. Qubit fluorometer (Thermo Fisher, catalog number: Q33238)
5. CFX96 Touch Real-Time PCR cycler (Bio-Rad, catalog number: 1855195)
6. 2100 Bioanalyzer (Agilent, catalog number: G2939BA)

## Software

1. FastQC (https://www.bioinformatics.babraham.ac.uk/projects/fastqc/)
2. Cutadapt (https://cutadapt.readthedocs.io/en/stable/#)
3. STAR (Dobin *et al.*, 2013)
4. Cufflinks (Trapnell *et al.*, 2012)

## Procedure

Notes:

1. For mRNA extraction frozen from stickleback gill tissue (~50 mg) use:
  250 μl of lysis buffer
  100 μl of Dynabeads
  600 μl of wash-A solution (two washes)
  300 μl of wash-B solution
  Elute to 20 μl RNase free water
  Volumes can be scaled in proportion for different size tissue samples.
2. Procedures B-G are adapted from the original Invitrogen Dynabeads protocol with modifications to solution volumes, sample homogenization and lysis steps. Additional steps H-J not part of original Invitrogen protocol are required to assure the yield of DNA-free and high-quality mRNA.
3. Procedures B-H should be followed through immediately. Safe stopping points where the experiment can be put to hold are after Procedures A and H.

- Dissect whole stickleback gills
  1. Euthanize fish following animal protocol guidelines (*e.g.*, EU Directive 2010-63-eu, and AVMA Guidelines) For example, by anesthetic overdose with NaHCO_3_-buffered Tricaine methanesulfonate (MS-222, pH 7.5). Note that, appropriate concentrations vary depending on the size of the fish. For sticklebacks we use a 10x solution (1.5 g/L) prepared by dissolving 1.5 g of MS-222 and 3.0 g of NaHCO_3_ in 1 L of aquarium water.
  2. Remove the head using scalpel and forceps (Figure 1). Working from the ventral side, open the operculum to reveal gill arches. Cut gill arches free from the ventral and dorsal attachment points as shown in Figure 1B. Remove any connective tissue that is not part of the gills.
  3. Collect gill tissue (~50 mg) in 1.5 ml Eppendorf Safe-Lock tubes and immediately snap-freeze in liquid nitrogen.
  4. Store samples in -80 °C.
    SAFE STOPPING POINT
    **Figure 1.**
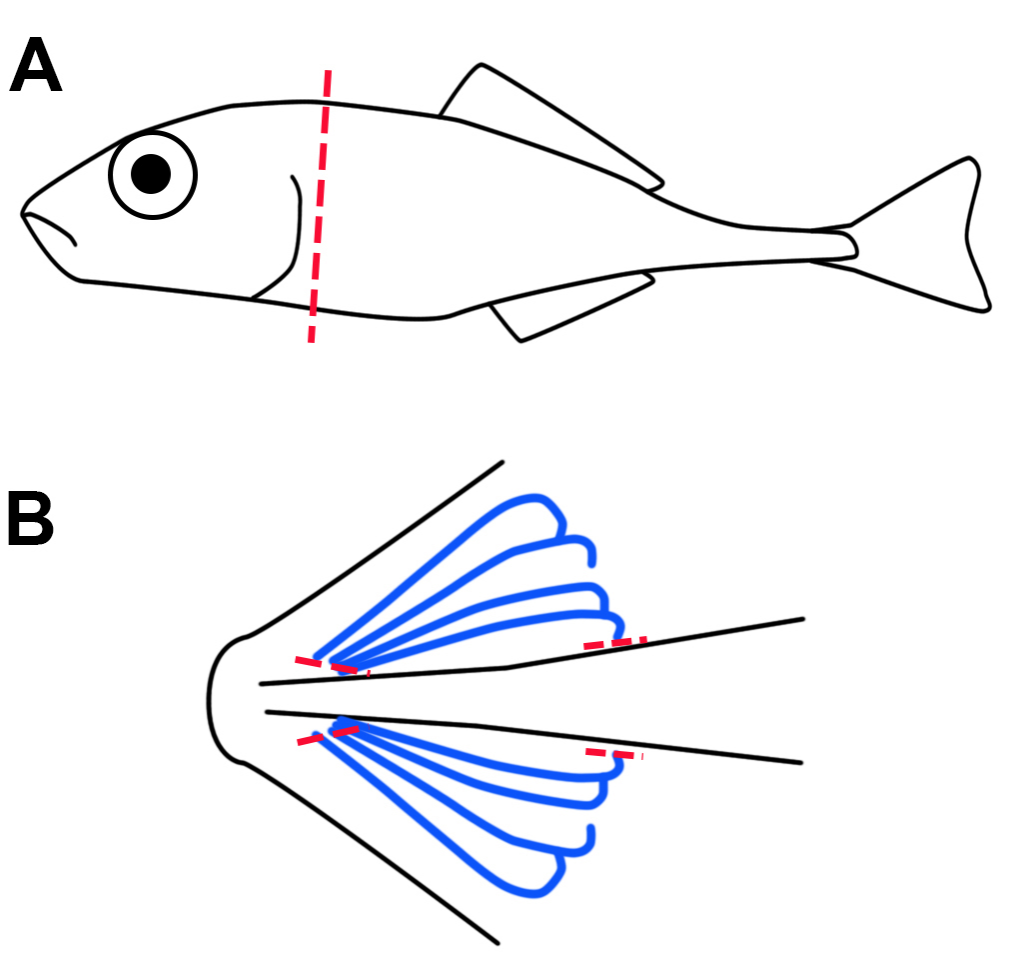
Dissection of stickleback gills. Whole gill arches are isolated from euthanized fish by removal of head via dissection along the transverse plane (red line, A). Then with a ventral view (B) the gill arches (blue lines) are excised by dissection of the ventral and dorsal attachment points (red lines, B).
- Wash and resuspend Dynabeads
  1. Allow Dynabeads and solutions to warm to room temperature.
  2. Prepare one 1.5 ml Eppendorf per tissue sample. Resuspend Dynabeads by gently pipetting up and down, and aliquot 100 μl of Dynabeads into each 1.5 ml Eppendorf tube.
  3. Place tubes on a DynaMag2 magnet and wait approximately 30 s for beads to form a pellet (~30 s).
  4. Remove the supernatant without disturbing the beads.
  5. Remove tubes from magnet and resuspend the Dynabeads in original volume of lysis buffer (100 μl).
- Homogenise tissue into a powder using a pestle
  Notes:
  1. Modify Micro-Tube homogenizing pestles for better sample disruption by cutting the round conical surface of the pestles into angular surface with sterile scissors or a blade (Figure 2).
    **Figure 2.**
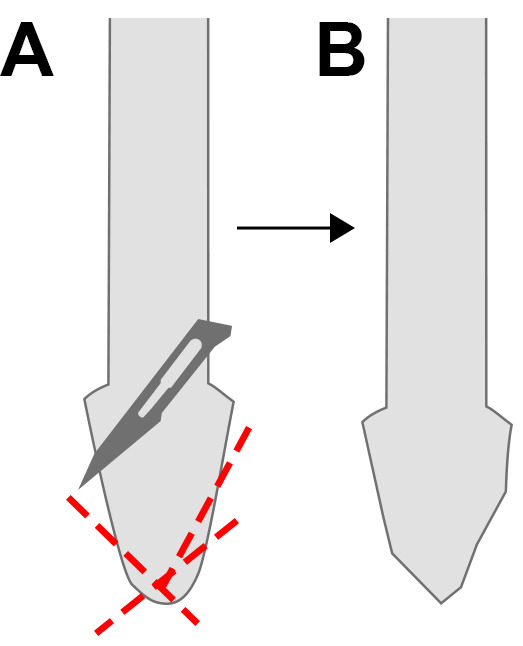
Modification of homogenizing pestle. For more efficient sample disruption, cut the surface of the pestle (red lines, A) into an angular surface (B) with sterile scissors or scalpel blade.
  2. Cool down the pestle before sample disruption by dipping it to liquid nitrogen.
  3. Remove samples from -80 °C and keep on liquid nitrogen.
  4. Disrupt tissue to powder on liquid nitrogen using a pestle stick in 1.5 ml Eppendorf tubes.
  5. Avoid melting of the sample at all stages before lysis step below. Freezing breaks the cell membranes and exposes RNA to endogenous RNases, which degrade RNA after thawing. Always keep your frozen sample on liquid nitrogen. Once you introduce an RNase inhibiting agent (for example LiCl in the Dynabeads lysis buffer, next step in protocol), RNA degradation is efficiently inhibited and sample can be thawed.
- Lyse the cells
  1. Take sample tubes one by one out of the liquid nitrogen, remove pestle but keep it in hand, immediately pipet in 250 μl of lysis buffer and then drop the pestle back into the tube.
  2. Keep the frozen tube in your hand and grind the frozen lysis buffer with the pestle until it melts to ensure the sample is suspended in lysis buffer immediately after thawing.
  3. Repeat for each of the samples.
  4. Discard pestle and shear DNA by passing the lysis mix five times through a 21 gauge needle before proceeding with the protocol.
  Note: Introduce lysis buffer while your tube is still frozen.
- Bind mRNA to Dynabeads
  Add 100 μl of washed and resuspended Dynabeads per sample, incubate 5 min at RT with continuing rotation (~500 rpm).
  Note: Continuous mixing during this incubation step is important to reduce the binding of Dynabeads to genomic DNA.
- Wash Dynabead-mRNA complex
  1. Place samples on Dynamag magnet and wait for beads to form a pellet (~30 s).
  2. Remove all supernatant from tubes without touching the bead pellet.
  3. Remove tubes from magnet and resuspend beads in 600 μl wash buffer A.
  4. Repeat Steps F1-F3 for a second buffer A-wash.
  5. Remove the buffer and perform final wash using 300 μl of wash buffer B.
  *Notes:*
  - Wash buffers should be room temperature before use.
  - Always resuspend the beads completely in the washing buffers by pipetting solution slowly up and down. Insufficient resuspension leads to increased DNA and ribosomal-RNA contamination. There should be no visible clump of beads after resuspension. Avoid excessive shearing/fragmentation of RNA by using the minimal amount of pipetting necessary to resuspend beads.
  - You can perform wash A three times for better sample purity.
- Elute mRNA from beads
  1. Place samples on magnet and wait for beads to form a pellet (~30 s).
  2. Remove all supernatant from tubes taking care not to leave any residual buffer.
  3. Remove tubes from magnet and resuspend beads in 20 μl of RNase free water.
  4. Incubate samples for 2 min in 65 °C in a thermocycler or thermomixer.
  5. Immediately place samples on the magnet and pipet out the supernatant to a fresh RNase-free 1.5 ml Eppendorf tube.
- Treat mRNA extraction with DNase
  1. To 20 μl of eluted mRNA, add 2 μl of Turbo DNase buffer + 1 μl Turbo DNase.
  2. Incubate at 37 °C for 20 min.
  3. Add 5 μl of inactivation solution, flick tubes and incubate 5 min at RT.
  4. Spin for 1 min 30 s at 10,000 *× g* and transfer the supernatant to a fresh RNase-free tube.
  5. Snap-freeze final mRNA extraction as smaller aliquots (*e.g.*, 10 μl) on liquid nitrogen and store in -80 °C.
    SAFE STOPPING POINT
  *Notes:*
  - Treat the entire 20 μl aliquot of mRNA with DNase right after elution from beads, do not freeze-thaw.
  - Either quantify and quality-control (e.g., Qubit & BioAnalyzer) your mRNA immediately after DNase treatment or keep an aliquot for these separately. Avoid freeze-thaw cycles of your mRNA sample at all times.
  - 20-30% ribosomal-RNA contamination of mRNA samples is common. rRNA contamination can be reduced by optimizing the A-wash volume. You can also try heating the lysis buffer to 65 °C or an elution-rebinding cycle in 65 °C while the beads are in the lysis buffer.
  - The Illumina TruSeq RNA-seq protocols start with mRNA selection, which eliminates the majority of ribosomal-RNA and DNA contamination (Figure 3).
    **Figure 3.**
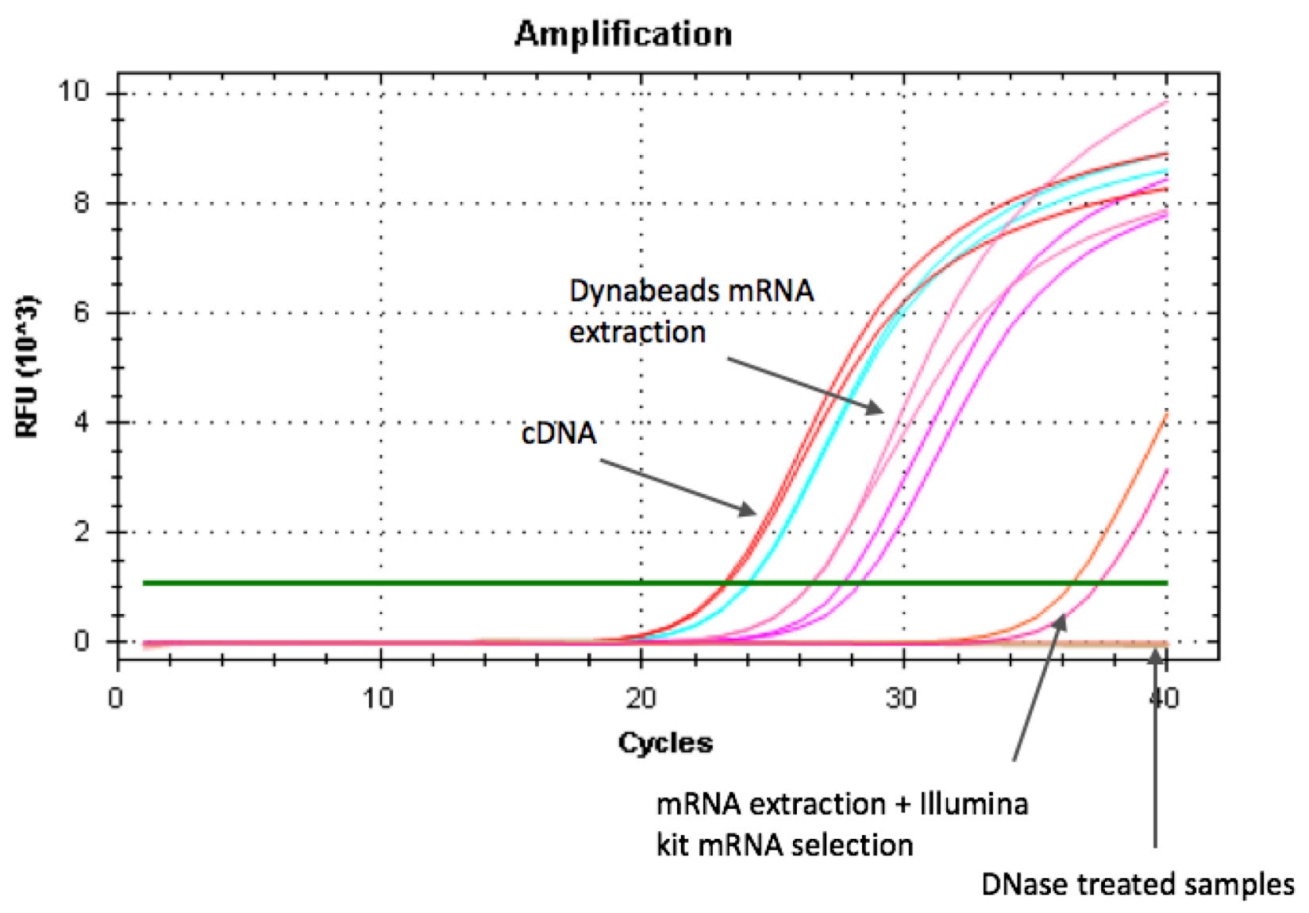
Testing for genomic DNA contamination in extracted mRNA using qPCR of housekeeping gene. Positive controls (cDNA) and mRNA extractions were amplified using qPCR of housekeeping gene primers (*e.g.*, claudin). Lower Ct values (PCR cycle when fluorescence can be detected above background level, green line) indicate higher abundance of template, which in the case of non reverse-transcribed mRNA extractions represents contamination from genomic DNA. Non DNase-treated Dynabeads mRNA extractions show DNA contamination, which is almost completely eliminated after second mRNA selection step performed at the beginning of Illumina TruSeq RNA-seq protocol. DNase treated mRNA extractions show no amplification and are thus free of DNA.
- Verify the absence of genomic-DNA contamination
  1. Design a pair of PCR primers that amplify a housekeeping gene (*e.g.*, claudin), making sure that primers amplify a fragment within a single exon (the PCR fragment should not span an exon-intron boundary). Aim for primers that are specific to the housekeeping gene (verify sequence similarity other genes e.g. using BLAST search), ending in G/C nucleotides and have a melting temperature of 60 °C ± 5 °C. For stickleback gill tissue, we used claudin gene as control (Fwd: 5' ACTTGGTGCCCTATCAAATGAGGTA 3', Rev: 5' AGTTATACACGACGGGAGGATTGAG 3').
  2. Select positive control samples and reverse-transcribe mRNA to cDNA using Takara PrimeScript RT Master Mix.
    - To 5 μl of mRNA extraction, add 3 μl of water and 2 μl of PrimerScript master-mix.
    - Incubate for 15 min at 37 °C.
    - Store in -20 °C.
  3. Prepare qPCR reactions for positive control (cDNA) and selected mRNA extractions for verification.
    10 μl of 2X Sybr select for CFX Master Mix
    1 μl of 10 mM Forward primer
    1 μl of 10 mM Reverse primer
    2 μl water
    1 μl cDNA template
  4. Run samples on qPCR using the following program:
    - 50 °C 2 min
    - 95 °C 2 min
    - 95 °C 30 s
    - 55-65 °C (primer-specific melting temperature) 30 s
    - 72 °C 30 s
    - Return to c. for 39 additional cycles
    - Hold at 4 °C
  5. Analyze qPCR results (Figure 3). The DNase treated mRNA extractions should not amplify, while the reverse-transcribed cDNA preps should give signal. Amplification in mRNA extractions without reverse-transcription indicates contamination from genomic DNA. In this case re-treat samples with DNase and verify mRNA quality using Bioanalyser.
- Verify the quality of extracted mRNA using Bioanalyzer RNA 6000 Nano reagents (Figure 4). For concentrations less than ~5 ng/μl use Bioanalyzer RNA 6000 Pico reagents.
  Note: RNA Integrity Number (RIN) typically calculated for total RNA extractions does not apply for mRNA extractions because the majority of ribosomal RNA used to calculate RNA quality and detect degradation have been removed. Instead, use the distribution of mRNA fragment sizes to determine whether the sample is of good quality (a range of sizes are of high molecular weight) or poor quality (highly degraded RNA of low molecular weight) (Figure 4).
  **Figure 4.**
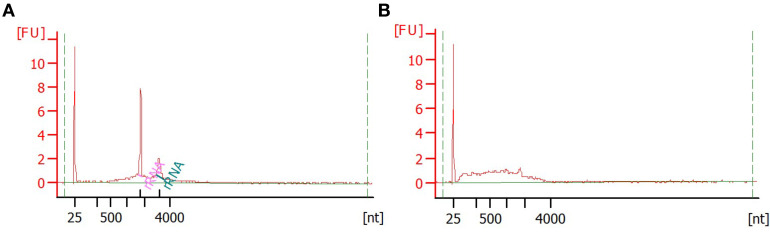
Verification of mRNA quality using Bioanalyser. Example of good (A) and bad (B) quality mRNA sample analysed with Bioanalyser RNA 6000 Nano reagents. Good-quality mRNA extraction can show some (10-30%) rRNA contamination. First peak at 25 nt corresponds to marker, two prominent peaks in (A) correspond to 18S and 28S rRNAs and the broad hump corresponds to mRNA. Note the left-shift (smaller size fragments) of the mRNA size distribution in the bad-quality sample, indicating that mRNA fragments are degraded.
- Construct RNA-seq libraries using Illumina TruSeq kit and kit instructions, with the following exceptions:
  1. Use 150 ng of mRNA as input (quantify using BioAnalyzer RNA Nano or Qubit RNA BR reagents). Dilute with RNase-free water to a final volume of 50 μl. Using adequate amount of input mRNA helps to avoid excessive PCR duplicates in final sequencing library.
  2. Optimize fragmentation time for the sequencing strategy you are going to use. Test the effect of fragmentation time using a time-series and analyze fragment sizes using Bionalyzer High Sensitivity DNA reagents, or refer to page 109 of Illumina TruSeq RNA Sample Preparation v2 Guide. For stickleback gill mRNA sequencing with HiSeq 3000 150 base-pair paired-end reads, we used two minutes (or 2 min) fragmentation to have a final average insert size of ~290 bp.
  3. Use provided indexes to identify samples after sequencing and pool in equimolar amounts for sequencing. Recommended strategy to avoid possible batch effects is to use as many indexes as there are samples and sequence all samples in the same lane. Where experimental design allows, use replicate lanes and combine reads from replicate lanes to reach the sequencing depth required.

## Data analysis

The following general example is given for 150 base-pair paired-end RNA-seq reads from gill tissue of threespine stickleback fish analyzed in a Unix/Linux/MacOSX environment with command-line tools. The user is encouraged to modify the paths outlined in the below procedure to match their data, and to execute the code in a terminal window by using shell scripts or copy-paste. Detailed information for the groups of samples tested can be found in Verta and Jones (2019).

1. Verify read and library quality with *FastQC* software following the guidelines outlined in *FastQC* website and manual. (http://www.bioinformatics.babraham.ac.uk/projects/fastqc/)
  Note: FastQC may report RNA-seq libraries to contain high levels of PCR-duplicates. This does not necessarily mean libraries have undergone excessive PCR because many reads are expected to map to highly abundant transcripts, which FastQC can interpret as PCR duplication.
2. Trim any Illumina sequencing adapters from the reads using *TrimGalore*. (http://www.bioinformatics.babraham.ac.uk/projects/trim_galore/)
  Note: Alternatively, quality-control and adapter trimming can be performed in one step using the software fastp (Chen et al., 2018).
  trim_galore \
  --path_to_cutadapt cutadapt \
  --illumina \
  --stringency 5 \
  --quality 20 \
  --output_dir out_dir \
  --paired \
  reads_R1_001.fastq.gz \
  reads_R2_001.fastq.gz
3. Generate *STAR* genome file (necessary only the first time a given reference genome used).
  STAR \
  --runMode genomeGenerate \
  --runThreadN N \
  --genomeDir genome \
  --genomeFastaFiles gasAcu1.fa \
  --sjdbGTFfile transcript.gtf \
  --sjdbOverhang 149 \
  --genomeChrBinNbits 18
4. Align RNA-seq reads to the reference genome (*e.g.*, Broad S1, gasAcu1 [Jones *et al.*, 2012]) with *STAR* aligner–a high performing aligner that addresses many of the challenges associated with aligning spliced RNAseq reads across intron/exon boundaries of the reference genome (Dobin *et al.*, 2013). Use manual two-pass mode for most sensitive detection of novel intron-exon boundaries.
  - For each sample, perform *STAR* 1^st^ pass to identify intron/exon boundaries in current sample.
    STAR \
    --readFilesCommand gunzip -c \
    --runThreadN N \
    --outFilterIntronMotifs RemoveNoncanonicalUnannotated \
    --chimSegmentMin 50 \
    --outFilterType BySJout \
    --alignSJDBoverhangMin 1 \
    --alignIntronMin 20 \
    --alignIntronMax 200000 \
    --alignMatesGapMax 200000 \
    --quantMode GeneCounts \
    --outWigType wiggle \
    --outSAMtype BAM SortedByCoordinate \
    --twopassMode None \
    --genomeDir genome \
    --readFilesIn reads_val_1.fq.gz reads_val_2.fq.gz
  - For each sample, run *STAR* 2^nd^ pass using intron/exon boundaries identified across samples above to inform read alignment.
    STAR \
    --readFilesCommand gunzip -c \
    --runThreadN N \
    --outFilterIntronMotifs RemoveNoncanonicalUnannotated \
    --chimSegmentMin 50 \
    --outFilterType BySJout \
    --alignSJDBoverhangMin 1 \
    --alignIntronMin 20 \
    --alignIntronMax 200000 \
    --alignMatesGapMax 200000 \
    --quantMode GeneCounts \
    --outWigType wiggle \
    --outSAMtype BAM SortedByCoordinate \
    --twopassMode None \
    --genomeDir genome \
    --limitSjdbInsertNsj 2000000 \
    --readFilesIn reads_val_1.fq.gz reads_val_2.fq.gz \
    --sjdbFileChrStartEnd SJ_1.out.tab \
    --sjdbFileChrStartEnd SJ_2.out.tab \
    …
5. Use *Cufflinks2.2* pipeline (Trapnell *et al.*, 2012) for reference-guided transcriptome assembly and transcript and isoform expression level testing. In order to run in “reference guided” mode, a transcript gtf file describing known gene models is needed. *Cufflinks* also allows “unguided” de novo transcriptome assemblies if this information is not available.
  cufflinks \
  -p N \
  --min-intron-length 20 \
  --library-type fr-firststrand \
  -o ./cloutGuided \
  --frag-bias-correct gasAcu1.fa \
  --multi-read-correct \
  --min-isoform-fraction 0.15 \
  --min-frags-per-transfrag 20 \
  --max-multiread-fraction 0.5 \
  -g transcript.gtf \
  reads.out.bam
6. Merge sample-level annotation files together (specified in assembly_list.txt).
  cuffmerge \
  -g transcript.gtf \
  -o merged_assembly \
  -s gasAcu1.fa \
  -p N \
  assembly_list.txt
7. Quantify RNA-seq read coverage over gene models.
  cuffquant \
  -o out_dir \
  -p N \
  -v \
  -u \
  --library-type fr-firststrand \
  -b gasAcu1.fa \
  merged.gtf \
  reads.out.bam
8. Normalize read coverages across samples.
  cuffnorm \
  -p N \
  -o out_dir \
  -library-type fr-firststrand \
  --labels sample \
  merged.gtf \
  abundances1a.cxb \
  abundances1b.cxb \
  abundances2a.cxb \
  abundances2b.cxb \
  …
9. Test for differential expression between groups of samples using the *Cufflinks* function *cuffdiff*.
  cuffdiff \
  -p N \
  -o outDir \
  -b gasAcu1.fa \
  -u \
  -dispersion-method per-condition \
  -library-type fr-firststrand \
  --labels Group1,Group2 \
  merged.gtf \
  abundances1a.cxb,abundances1b.cxb \
  abundances2a.cxb,abundances2b.cxb \
  …
